# The Hospital. Nursing Section

**Published:** 1903-10-17

**Authors:** 


### The Hospital.

### Nursing Section.

Contributions for this Section of "THE HOSPITAL" should be addressed to the EDITOR, "THE HOSPITAL" NURSING SECTION, 28 & 29 Southampton Street, Strand, London, W.C.

No. 890.—Vol. XXXV. *SATURDAY, OCTOBER* 17, 1903.

### Notes on News from the Nursing World.

### OUR CHRISTMAS DISTRIBUTION.

Time passes so swiftly that our readers who kindly intend to help us with contributions to our Christmas distribution of clothing will pardon us for reminding them that there are only two months between this and the date of our second exhibition of garments made or purchased for the use of hospital and infirmary patients. However great may be the number of contributions, we gather from the urgent appeals we have received for a share of them, that they will fall short of the needs of the sick poor. All parcels should be addressed to the Editor, 28 & 29 Southampton Street, Strand, London, W.C., and should have "Clothing Distribution" written outside them.

### QUEEN ALEXANDRA'S MILITARY NURSING SERVICE.

On Saturday Lady Roberts, accompanied by Miss Sidney Browne, matron-in-chief of Queen Alexandra's Imperial Military Nursing Service, visited the Princess Louise Hospital at Alton, for the purpose of presenting badges of the service to the members of the nursing staff. The proceedings were private. The provisional appointments of Miss L. E. Mackay, Miss E. L. C. Crowe, and Miss K. M. Hewetson as staff nurses of the Military Nursing Service are gazetted.

### MR. ALFRED LYTTELTON AND COLONIAL NURSING.

There is reason to believe that the new Secretary of State for the Colonies will adopt the same attitude with regard to the Colonial Nursing Association which was pursued by his distinguished predecessor, and that the sympathetic support of Mrs. Alfred Lyttelton may also be counted upon by the managers, who will, no doubt, invite her to become a member of the council.

### VICTORIAN TRAINED NURSES' ASSOCIATION.

The second annual meeting of the Victorian Trained Nurses' Association was held at the Athenæum Hall, Collins Street, Melbourne, on Thursday, August 27th. The president, Dr. Springthorp, presided. The report and balance-sheet were passed, and printed copies of the suggested rules for the Benevolent Fund were circulated for approval. Two examinations have been held by the Conjoint Board of Examiners in Melbourne, Ballarat, and Bendigo. In the first all passed, in the second 21 were successful and seven failed. A number of private hospitals which employ only certificated nurses have been registered, together with ten nurses' homes. A suggestion was made by the president that there should be a special examination for nurse wishing to qualify for the position of matron. The question of the constitution of a board of examiners was not gone into. It is most essential that where small country hospitals are of necessity training schools the matron should be not only an expert nurse, but a capable teacher, housekeeper, and organiser likewise. Doubtless full consideration will be given to the subject before it is brought before the council, but it is anticipated that there will be serious difficulties.

### THE NEW BISHOP OF MANCHESTER AND NURSING.

Nurses in the diocese of Manchester will be glad to know that the new Bishop, Dr. Knox, is a warm and judicious friend of the nursing movement. While he was Bishop Suffragan of Coventry he founded, in addition to the Ellen Knox Memorial Homes—established as a means of providing a meeting-place for girls and others employed in factories during the day, who at night have to seek amusement outside their own homes—a parochial nursing institution. The staff consists of three trained nurses, and several probationers whose duties are to attend midwifery cases and other patients who, being unable to remain in hospital, are not in a position to secure proper nursing.

### THE NURSES OF MOUNT VERNON HOSPITAL.

A distribution of prizes and certificates to the nurses who had successfully passed the various examinations took place last week at the Mount Vernon Hospital for Consumption and Diseases of the Chest at Hampstead. Afternoon tea was served in the nurses' new dining-room, after which the members of the committee and the nursing staff assembled in the adjoining sitting-room. Six nurses entered for and passed the final examination qualifying for the certificate of two years' training, and six received certificates for theoretical and practical dispensing and for massage. First and second prizes of medical and surgical books, given by the chairman of the House Committee, were awarded to Nurse Yates and Nurse Gertrude Phillips, who had the highest total marks in their final examination, and also for ward work and general conduct during their two years' probation. Additional prizes were also awarded in various special class examinations, and Nurse McFarlane took the first year nurses' prize for general efficiency. This is the first time that certificates have been given on the completion of the two years' course of training, which now includes nursing and bandaging classes, lectures on anatomy and physiology. A

four-months' course of theoretical and practical dispensing and a course of instruction in massage is given by the matron, who is a certificated masseuse.

### SUPERFLUOUS NURSES AT THE ASYLUMS BOARD HOSPITALS.

It is very essential that the managers of the Metropolitan Asylums Board should have a sufficient reserve of nurses to cope with any sudden increase in the number of patients at the acute fever hospitals; but it is also important that they should not waste the money of the heavily-burdened ratepayers. In March, 1893, they decided that the managers "should, from time to time, fix a minimum below which the nursing staff shall not be reduced, and that for the present such minimum be the number required to staff the permanent wards in all the acute fever hospitals, and to provide for the performance of the ambulance service." This resolution has just been rescinded, and the facts cited by Mr. Helby justify the assertion that the managers have not shown the same regard for the pockets of the public as they would probably have manifested if they had been required to pay the piper themselves. According to Mr. Helby's statement the other day there were at the beginning of October 157 patients in the South-Western Hospital, and 266 members of the staff; 301 patients at the Grove Hospital, and 300 members of the staff; 215 patients at the South-Eastern Hospital, and 221 members of the staff; 238 patients at the Park Hospital, and 257 members of the staff. These figures prove that the minimum staff has been considerably larger than the circumstances require. If this state of things has been going on for any length of time the managers have been guilty of a serious derogation of duty in maintaining a number of superfluous officials.

### ONE NURSE TO FORTY PATIENTS.

The report of the workhouse committee of the Upton-on-Severn Union, who have had under consideration certain proposals for improving the nursing arrangements at the infirmary, has been adopted by the guardians. The chairman informed his colleagues at the last meeting that the need for improvement had been impressed upon the guardians of the Local Government Board, and when it is added that there has until now been one day nurse and no wardmaid for nearly forty patients, it will be a matter of astonishment that the need was not recognised before. However, in future there are to be two day nurses and one night nurse, with a wardmaid. In view of the chairman's statement that there are frequently difficult cases to deal with, the increased staff is certainly not in excess of the requirements of the patients.

### PAUPER NURSING DIFFICULTIES.

At the South Western District Poor Law Conference at Exeter, last week, a paper on "Pauper Nursing" was read by the Rev. F. F. Buckingham, Chairman of the St. Thomas Union. He observed that he should like to have seen some kind of recommendation on the Departmental Committee report laying down a maximum and minimum proportion of nurses to patients to check needless extravagance on one hand and parsimony on the other. He considered that a thoroughly competent staff of nurses was a necessity in all workhouses, and to obtain them the Guardians must offer adequate salaries, study their comfort and convenience, both on and off duty, provide sufficient recreation and liberal leave. The Vice-Chairman of the Salisbury Board (Mr. Hammick) contended that it would be impossible to lay down any rule as to the number of nurses to be employed, and urged that what was wanted for Workhouse Infirmaries was not so much nurses who had been through the operating theatre as those who were bubbling over with the milk of human kindness, were competent and sympathetic. Dr. Waylen, Medical Officer at Devizes, contended that if Unions treat and pay their nurses well, and give them plenty of liberty there will be no lack of applications. The general conclusions were that as Workhouse nursing does not offer the same scope for advancement as nursing in general hospitals, greater inducements will have to be offered to obtain and retain nurses, such as increased salaries, more freedom, better comforts, less menial duties, and a pension at an earlier age than is at present conceded.

### A NEW INSTITUTE FOR THE TRAINING OF NURSES.

Under a scheme which has been proposed by the Charity Commissioners for the consolidation and future administration of some of the principal charities belonging to the parish of St. Mary, Newington, the sum of £500 a year out of some 14 foundations for the benefit of the local poor is to be set apart for "the provision of nurses for the sick and infirm, for the benefit either of the poor of the parish generally, or of such deserving and necessitous persons resident therein as the trustees select for this purpose, and in such way as they consider most advantageous to the recipients, and most conducive to the formation of provident habits." The trustees are also to be empowered to expend £3,000 "in building and furnishing an institute for the training of nurses."

### OPENING OF THE MEMORIAL HOME AT RIPON.

The new Nurses' Home which has just been opened at Ripon as a memorial of the golden wedding of the Marquis and Marchioness of Ripon, is a handsome building with a pleasant garden in front. The cost of erecting it was £1,600, and this together with the sum of £250 for furnishing, has been subscribed by the people of Ripon and the locality. The ceremony at the opening was of a very interesting character, Lord Mountgarret, in asking Lady Ripon to accept the key prepared for the occasion, and to do the trustees the honour of opening the door and taking possession on behalf of herself and Lord Ripon of the Nursing Home, said that the presentation was a mark of gratitude for the two noble lives that had been lived near Ripon for the past fifty years. After a memorial tablet had been unveiled, and Lord and Lady Ripon had been conducted over the Home, the matron presenting Lady Ripon with a bouquet, Lord Ripon formally handed the building over to the Ripon Nursing Institution. He expressed the gratitude of Lady Ripon and himself for the splendid present, and said it was their wish that their golden wedding should be celebrated by something which would be of permanent utility to the citizens of Ripon and the inhabitants of the neighbourhood.

### DARLINGTON QUEEN'S NURSES' ASSOCIATION.

A copy of the eleventh annual report of the Darlington Queen's Nurses' Association has been sent to us by the hon. sec. Mrs. Arthur F. Pease, from which we learn that the work was carried on during the twelve months in a quiet and beneficial manner. As the result of several special entertainments in aid of the Association the adverse balance of £70 odd, with which the year began, was by December somewhat reduced; but the committee are naturally disappointed that it should still, after eleven years of good work, be necessary to raise funds by such methods. They have certainly a right to expect that the needs of the organisation, which are under £500 a year, should be fully met by subscriptions. This, seeing that in the twelve months between 14,000 and 15,000 visits were paid and nearly 500 cases attended, is a moderate sum, and cannot be beyond the means of Darlington to find in the ordinary manner. We hope that the Darlington Association will soon have as large a balance in hand as the District Nursing Institute at Darlaston, which by a mistake in our issue of August 29th was associated with Darlington.

### NURSES AND BICYCLES.

With reference to our remarks a fortnight ago on the results of Miss Greg's offer to give a discarded bicycle to a district nurse, we have received a letter from a nurse in Gloucestershire who says that, as she has a large and scattered district, she would be very grateful if we "could procure" her a bicycle. We should be only too glad if we could procure bicycles for all district nurses who have small salaries and large districts, but we have no fund for such a purpose, and we are afraid from Miss Greg's letter that she already has a great many more applicants than she is likely to have bicycles.

### SUCCESSFUL PROBATIONERS AT BRADFORD INFIRMARY.

In connection with the recent examination of probationers in medical and surgical nursing at Bradford Royal Infirmary, the Mayoress of Bradford presented Nurse Hall with the gold medal, and Nurse Willis with the silver medal. In the first division a high number of marks were also attained by Nurses Morley, Menzies, and Hay; in the second by Nurses Williamson, Mackenzie, and Cranswick; and in the third by Nurses Williams, Harbottle, and Harrison. Prior to the presentation the Mayor referred to the departure of the late matron for Johannesburg, and said that in Mrs. Magill the board had been fortunate in finding a successor. There are now 50 probationers in training in the infirmary.

### THE TESTIMONIAL QUESTION.

The Southampton Guardians have had a discussion on the proposed rescission of the resolution instructing the medical superintendent and the matron of the Shirley Warren Infirmary not to give testimonials or references to any of the staff save those that are made out on a printed form and issued from the clerk's office. The chairman having maintained that, in the light of a recent event—to which we have already alluded—the original resolution was necessary in the interests of justice and equity, a division was taken and the proposition to reverse the policy previously decided upon was defeated. It may be hoped that now the matter has been thoroughly threshed out the new and quite equitable arrangement will be acquiesced in even by those who prefer the method which caused mischief.

### THE GRANARD SCANDAL.

The Granard Board of Guardians persist in their attitude of defiance to the Irish Local Government Board. Their latest action has been to pass a resolution calling upon Dr. Kenny to apologise to the nurses lately employed as nurses in the infirmary for any offences which he may have given them during the unhappy relations which have existed between them, and for the second time to reappoint the master who has already been dismissed by the Local Government Board. Nearly all the speakers at the last meeting adopted a tone of hostility to the Board, and maintained their right to independent action. Mr. Patrick Quinn had, indeed, the good sense to emphasise the fact that the ratepayers must suffer by the continuation of the quarrel, but Mr. Donlon appeared to express the almost universal feeling of the guardians when he declared that "they would not knuckle down to the Local Government Board on this point."

### BELFAST "NURSES' OWN."

The second monthly gathering of the "Nurses' Own" was held on the first Thursday in October, when there was an attendance of about 80 nurses from the different hospitals and homes. After tea solos were contributed by Nurse Donaghy, Miss Cairns, Nurse Douglas, Mr. R. K. McClean, and Mr. A. J. Downham. Short addresses were also delivered by Mrs. Hoggard and Pastor Gunn. Each nurse, before leaving, was presented with a bunch of flowers, provided by some friends, whose thoughtfulness and kindness were much appreciated by the recipients. These monthly meetings were organised by Mr. A. J. Downham, and they are the only gatherings of the kind in Ireland.

### SHORT ITEMS.

The Incorporated Society of Trained Masseuses, 12 Buckingham Street, Strand, will hold an examination on Tuesday and Wednesday, November 24th and 25th. Particulars can be obtained from the Secretary. No application can be received after November 2nd.—The collections this year at the annual demonstration organised by the Woolwich and District Trade, Friendly, Temperance and other societies on behalf of the Woolwich, Plumstead, and Charlton Nursing Association amounted to £47, or £11 more than last year.—The usual winter entertainments organised for the in-patients of the Cancer Hospital, London, commenced on Thursday last week, when Mr. Laurie Phillips provided an enjoyable programme which was greatly appreciated by all present.

### Lectures on Ophthalmic Nursing.

By A. S. Cobbledick, M.D., B.S.Lond., Senior Clinical Assistant and late House-Surgeon and Registrar to the Royal Eye Hospital.

### LECTURE XXI.—CATARACT (*cont.*).—THE PROGNOSIS, DIAGNOSIS AND TREATMENT.

*Prognosis.*—(i). *As to the length of time a senile cataract will take to mature.* This varies very much in different individuals, and an opinion should not be too confidently expressed before the case has been watched for about three months. Speaking broadly, it may be said that those which commence at the periphery mature more quickly than the nuclear variety. Diabetic cataracts often mature rapidly. Traumatic cataracts become opaque very rapidly. Congenital cataracts do not become more opaque.

(ii). *As to the vision that will result after extracting the cataract.* This depends on three factors: the condition of the fundus, the possibilities of accidents—such as hæmorrhage or considerable loss of vitreous—at the time of operation, and the amount of débris which is left behind. It is obvious that if there is disease in or about the retina of the eye affected with cataract, the extraction of the cataract may not produce much improvement in vision, but considerable disappointment to the patient.

If the cataract is seen in an early stage, the condition of the fundus may be noted and the probable result of a later operation may be safely prognosed; if, however, both eyes are so affected with cataract that the fundi are obscured, a rough test must be used; this is termed the *light projection,* and consists in the ability or inability of the affected eye to determine from which direction rays of light are thrown on to the eye. If the patient finds no difficulty in localising the light the projection is termed good and the probabilities are that there is a normal fundus behind the cataract; if the patient cannot localise the position of light, the projection is bad and indicates a diseased fundus, and probably but slight improvement after an operation. The second factor need only be suspected where there is some constitutional disorder or extreme nervousness on the part of the patient at the time of operation so that self-control is lost and possibly some vitreous expressed through trying to close the eye after the section has been made.

The debris which remains after an extraction, termed the secondary cataract, varies in amount: the capsule of the cataract is, in the usual operation, always left behind with a varying quantity of cortical substance, the amount of the latter partly depending on the maturity of the cataract and partly on the dexterity of the operator.

If a congenital cataract in a young child is not operated on for some years, the result will not equal that obtained by early operation as the function of the eye becomes impaired from disuse.

*Diagnosis.*—Patients with early cataract complain of inability to be properly suited with glasses: dark specks in front of the eye which move only when the eye moves, and slight diplopia are also common complaints. As long as one eye retains good vision there is usually little complaint, but as soon as the sound eye becomes affected the presence of cataract is discovered. The condition may be suspected when an individual over 55 or 60 years of age has failing distant vision, which is worse in one eye than the other, but no diagnosis of an early case can be certain before the pupil has been enlarged by the action of a mydriatic, *e.g.* a drop or two of Gutt homatropine gr. iv. ad. ℥ i., then, by reflected or oblique light the fine striæ commencing at the periphery are at once evident.

If the vision is very much impaired and the cataract only in an early stage of development, some fundus trouble must be suspected and searched for.

*Treatment.*—No remedy in the form of drugs taken internally or used externally is known to have any effect in retarding the growth of or in maturing a cataract.

People with central opacity of the lens may derive rather better vision by the continued use of a mydriatic, *e.g.* atropine sulph gr. i. ad. ℥ i.

Cataracts in children and young adults, in whom no hard nucleus has developed, are treated by the method of *discission,* the lens capsule is perforated with a needle and its substance broken up; the result is that the aqueous gains entry and becoming absorbed, so that the lens substance swells and becomes opaque; after a time much of it becomes absorbed; if it does not absorb, or if the tension of the eye becomes much raised, the lens substance is evacuated through a small incision in the cornea by means of a grooved evacuator. Evacuation is often most necessary in the treatment of traumatic cataracts or after needling them they rapidly become opaque and swell to such an extent as to press forward the iris and obliterate the infiltration angle and so stop the normal circulation of the aqueous, causing a consequent rise of tension and the necessity of early evacuation.

Cataracts in individuals over middle age must be extracted in toto, when they have become mature. There are several modes of procedure:—

1. An iridectomy may be performed a month or two before the extraction is attempted.
2. An iridectomy may be performed immediately before the extraction.
3. The cataract may be removed without an extraction, so leaving the iris quite intact.

The advantages of performing a preliminary iridectomy (1) are:—

*a.* If the cataract is slow in maturing, this operation often hastens the process.

*b.* As these operations are always performed under cocaine anæsthesia, the surgeon obtains some idea as to the probable amount of self-control the patient will exhibit when the more difficult operation of extraction is performed, and will consequently be prepared for any emergency.

*c.* That the extraction is not only rendered more easy but is less likely to be followed by any inflammatory trouble.

*d.* If the tension is raised there is less tendency to lose vitreous when the extraction is performed.

The disadvantage is that two operations are necessary.

Extraction without an iridectomy is a more difficult operation, prolapse of the iris is not uncommon, and there is more likelihood of loss of vitreous. The advantage is that the pupil remains unaltered.

### To Nurses.

**We invite contributions from any of our readers, and shall be glad to pay for "Notes on News from the Nursing World," or for articles describing nursing experiences, or dealing with any nursing question from an original point of view. The minimum payment for contributions is 5s., but we welcome interesting contributions of a column, or a page, in length. It may be added that notices of appointments, entertainments, presentations, and deaths are not paid for, but that we are always glad to receive them. All rejected manuscripts are returned in due course, and all payments for manuscripts used are made as early as possible after the beginning of each quarter.**

### Irish Dispensary Midwives and their Work.

BY A BRITISH NURSE.

Now that the Midwives Act will soon begin to take practical effect in England and Wales I notice a desire among those workers who are most concerned to gain more knowledge of the systems adopted in other countries far and near. As we all require to gain added experience on this subject, it will, I think, be of interest if I summarise briefly a few points regarding the plan of dispensary midwives in Ireland which has for some time worked in a very satisfactory manner.

The result aimed at in this system is that the poor should not suffer neglect owing to any avoidable difficulties or misunderstandings between doctors and midwives. The rules laid down by the Irish Local Government Board are exact and comprehensive, and they clearly define the respective duties of the medical officer and midwife. Although we may not desire or need to adopt the scheme in England, it is a very valuable one to study, suggesting as it does a workable form of machinery. The following is a brief summary of the system regulated under the Irish Local Government Board.

The medical officer is not relieved from his responsibility by the fact that a midwife is officially employed in the district; it is distinctly stated that her services are placed at his disposal to relieve him from the necessity of attendance in cases of natural labour. On receiving a "ticket" for attendance he must ascertain by visiting the patient the nature of the case. If he finds it is one likely to be attended with any difficulty or danger he will take charge of it and may also requisition the service of the midwife.

If he is satisfied the case is normal, it is only needful for him to place the midwife in attendance, directing her to send for him if needful. Tickets for attendance may be sent to the medical officer or the midwife at the option of the patient; this seems a fair arrangement, as the patient may prefer the services of a competent woman.

A dispensary midwife is appointed by the Board of Guardians, and her salary is paid by them.

She must have obtained from some recognised lying-in hospital a certificate as to proficiency in midwifery, unless she has held office previously as midwife to a workhouse or dispensary, for which appointment she was at the time qualified. Her age must be over 25 years.

She is allowed to take cases, and receive a fee, outside her parish work, provided that such cases do not interfere with her duties as dispensary midwife, which have the first claim on her services. She may be called in by the medical officer, or requisitioned by the patient on a ticket signed by a Guardian, a warden, or the relieving officer.

It is necessary for the midwife to reside in a convenient place in the district, within easy access of the medical officer, and she must execute all his lawful orders which are applicable to her office.

She must punctually attend all cases of midwifery to which she is called, and continue in attendance on each patient until her services are no longer necessary.

On the termination of cases where a medical officer has not been called in, the midwife must report the result to him. She must keep a register of all cases she attends, and must submit it monthly to the Guardians for examination. As midwives may take other than Poor Law cases they are not wholly dependent on their official salaries, which are now usually about £30 a year. It should be borne in mind that life in Ireland is not so expensive, especially in the country, as it is here.

There are over 550 midwives employed in the dispensary districts throughout Ireland; but unfortunately, the Local Government Board do not publish statistics showing the number of cases attended annually by them. If in any case attended upon a "ticket" the midwife finds it necessary to call for the assistance of the medical officer, and intimates the same to him, he must immediately take charge of the case. Cases of abortion and miscarriage are to be regarded as midwifery cases.

The accompanying ticket is sent to a midwife when her attendance is required:—

*Form E.* 3 (*with Counterfoil*).

TICKET FOR ATTENDANCE OF MIDWIFE AT THE PATIENT'S HOME.

To Midwife of
Dispensary District, in Union.

MADAM,

You are hereby directed to visit at once residing at
in the above Dispensary District, who is by occupation
a , and afford her the necessary attendance and nursing.

Dated this day of 1 .

(*Signed*)

*Guardian, Relieving Officer*, or *Warden*, as the case may be.

Through the action of the Local Government Board of Ireland, the midwife has held for years a more definite and therefore better position than has the same class in England; that is, her position has been recognised officially, her qualifications clearly stated, and her duties exactly determined in the general regulations. It is not surprising that this being the case the principal Dublin lying-in hospitals insisted during the passage of the bill that their certificates should be recognised as qualifying for registration under the Act. Midwives who work as monthly nurses in England may enjoy higher fees, but they have not the same recognised position as the dispensary midwives in Ireland. The latter class is united under a department which fully recognises their value as workers.

### First Night in a Private Nurses' Home.

BY A SOMETIME INMATE.

I HAD just finished my hospital training and had decided to adopt private nursing, accordingly I joined a nurses' home in one of our large towns. After a well-earned holiday I left for the South by a midday train, arriving at my destination early in the evening. A friend met me at the station, and we took a cab to the home. I had barely time to give her some scraps of home news when the cab stopped and we both got out. I rang the bell which was answered by a smart maid and we stepped into a large hall, the cabby carried in my box, my friend bade me goodbye, and I was left standing alone (the maid had vanished), bag in one hand, umbrella in the other, and in all the glory of a new uniform to which I had not yet got accustomed.

THE MATRON.

I seemed to stand there hours—in reality minutes—and to pass the time I read some rules for nurses, which were printed in large type and hung in a place of vantage. Meantime nurses were going out and in, giving me a passing glance as they went, and one of them addressed me with "Hullo, Nurse C.!" and then, seeing that she had made a mistake, she begged my pardon and passed out. Just then a side door opened and a pretty, small, middle-aged lady, whom I at once recognised as my future matron, came out, and catching sight of me, remarked: "Oh, Nurse B., is it you?" "No," I answered desperately, wondering how many more I resembled, and not wishing to let the opportunity pass, "I am Nurse F., and I've just arrived." "Oh, how d'ye do, I thought you were someone else in the shadowed light," and, moving towards a door, she led me into a large room—a dining-room. She brought me a chair by the fire, and she sat down in another and chatted brightly with me. After we had discussed business matters, she left in order to send me some tea. Tea arrived, and as I drank it I felt my spirits rise and began to feel interested in my surroundings. The furniture was plain and substantial, a few good pictures hung on the walls, and plants in pots, and some cut flowers relieved the sombreness of the room.

### An Introduction to my Bedroom.

Just as I had finished tea a sweet-looking nurse came in and said she had been told off to take me to my room. Following her up two flights of stairs she ushered me into a large airy bedroom containing two beds, one of which I was told was occupied at present by a Nurse M——. I changed my dress, donned cap and apron, and all the time nurse chatted and told me many things about the home. Then we went down to the general sitting-room in which there were about a dozen nurses all occupied in different ways. One or two were sewing, one was reading, one was writing letters, but the chief attraction seemed to be centered in one who was making sweets at a side table. I was introduced all round as the "new nurse," and then I sat down beside the one who had been writing, but who seemed now to have finished, and to be ready to talk.

### Sitting-room Conversation.

We found we had some things in common. She appeared to be about the same age as myself, (she was also "new," having arrived two days before, she was recently trained, and she was longing for a first case. She told me tit-bits about some of those present—how the pale-faced nurse was recovering from a recent illness, how another had just come in from a case abroad (we wished we'd get one abroad), and how the one with the tanned skin had lately come back from India, where she had been for two years. As we talked scraps of conversation reached us: "We three have got late leave for the theatre, may I have your lace ruffle, Eva?" "It is getting rather dirty with so much lending, but you may have it with pleasure," was the rather weary answer. From another quarter: "Yes, it was a climb down. I had left one case and had been driven to the station in a luxuriant carriage drawn by a pair of bays, and on going to my next case I found a farm-cart had been sent to meet my train, and I was asked whether I preferred to walk or to take a seat on my box in the cart. Needless to say I walked." Then the sweets were sampled and I was asked if I would have one, and so the time passed on pleasantly till the clock struck eight.

### Supper and Bed.

Then the supper-bell rang. We all went down, and in the dining-room I saw some more nurses whom I had not seen before. After supper, which consisted of coffee or cocoa, cold meat, etc., came prayers, and then we all went off to our respective rooms. My companion of the evening turned out to be Nurse M. On reaching our room we sat down and talked again. "This room reminds me of a prison," said nurse. It certainly had a bare look, and the red quilts on the beds, I daresay suggested the thought to her. Shortly after we got into bed, each wondering what the morrow would bring forth, "There's a dead-awakening bell rings at 7.15 and the cabs rattle all night on the street, so I hope we'll sleep," said a muffled voice from the other bed.

### My First Case.

It certainly seemed no time till the awful reveillé sounded and banished sleep for ever. We got up, dressed quickly, and went down to partake of a tempting breakfast with the same nurses who were there the previous night. After breakfast I was rather surprised to be told by matron that I was to go to Paisley to help another nurse at a case there. I did the little packing I had to do, and, dressed and ready, went to the sitting-room to await the arrival of my cab. Nurses here were sent to cases, not in the order that they came in, but according to the matron's choice. Amidst many expressions of good luck, "I wish I was going," "You'll find Nurse N. very nice," etc., I took my departure, and went away to my first case.

### Hints on How to Start a District Nursing Association.

(*Continued from Page* 29.)

The village nurses, however, do excellent work, and especially in maternity nursing are a very great improvement upon the ordinary village "Gamp," and can do much to banish the harmful practices so frequently resorted to in the treatment of mother and child. But there should certainly be some system of inspection to keep the nurses up to the mark. While at the training home they are under the strictest supervision, and are scarcely allowed to do anything on their own responsibility. They are looked after by superintendents and senior nurses, and there are doctors all round to come to their aid if needed. Contrast this with the position of a village nurse when placed in a country district to begin the work of a District Nursing Association. She has to work single-handed now; there may be a doctor in the place, or there may not be one nearer than three or more miles away; all strict supervision of her work is removed, and she is now under an unprofessional committee who cannot go with her to her cases like her former superintendents, and who would not know if she were doing her work as it should be done even if they could. Very often too, there is not enough work to keep her busy, or what there is lacks the interest of the work while at the training home in London or some large town. Time hangs heavy on her hands, and she is apt to become lax and careless, there being no means of keeping her up to the standard even of her short training. This want, however, is being supplied in many parts of the country by means of affiliation with County Nursing Associations, which offer free training as village nurses to suitable candidates, and appoint superintendents who are trained nurses to visit the affiliated districts at regular intervals. In this way the nurse is not only encouraged and helped, but her training is continued.

### Lodgings for the Nurse.

Some suitable place will next have to be found where the nurse may live. As a rule a district nurse in a country town or village lives in lodgings which should consist of a sitting-room and bedroom, and should be as comfortable as possible

### HINTS ON HOW TO START A DISTRICT NURSING ASSOCIATION—*Continued.*

and in a central position. She may be allowed to choose her own lodgings, but the committee can generally help her to find them. The price of lodgings varies according to the style of rooms required and the rate of terms in the place. Sometimes a cottage is taken for the nurse, but this is not to be recommended unless the nurse has someone to live with her and relieve her of the housework and cooking, etc., and see that she gets her meals regularly. Otherwise she will be working when she ought to be resting, and often when coming in tired will make any sort of a meal do instead of the substantial one she needs—the result being a frequent breakdown.

### The Salary Question.

There is a wide range of salary for district nurses, which roughly may be anything from about £40 to £100 per annum inclusive, according to the kind of nurse employed. An inclusive salary covers the expense of board, lodging, laundry, and uniform, but sometimes a certain salary is offered, with rooms or uniform. Thus in one case the arrangement may be "salary £80 inclusive," in another "£50 and rooms," in a third "£35 with board and lodging." The following examples may serve—No. I. as an approximate maximum, and No. II. as an approximate minimum:—

I.

| | £ | s. | d. | |
|---|---|---|---|---|
| Salary ... ... ... | 35 | 0 | 0 | per annum. |
| Board, 10s. per week ... | 26 | 0 | 0 | „ |
| Lodging, 10s. „ ... | 26 | 0 | 0 | „ |
| Laundry, 2s. „ ... | 5 | 4 | 5 | „ |
| Uniform ... ... ... | 5 | 0 | 0 | „ |
| | £97 | 4 | 5 | „ |

II.

| | £ | s. | d. | |
|---|---|---|---|---|
| Salary ... ... ... | 15 | 0 | 0 | per annum. |
| Board, 6s. per week ... | 15 | 12 | 0 | „ |
| Lodging, 4s. „ ... | 10 | 8 | 0 | „ |
| Laundry, 1s. „ ... | 2 | 12 | 0 | „ |
| Uniform ... ... ... | 2 | 0 | 0 | „ |
| | £45 | 12 | 0 | „ |

Village nurses receiving free training under a County Association agree to work so many years at a stated wage, the minimum being about 14s. per week, or its equivalent, and uniform inclusive. There are other incidental expenses —such as nursing requisites and appliances, extra or temporary nursing help, printing expenses, hire of room for annual meeting, etc., which should be taken into consideration when calculating the cost of supporting a district nursing association.

### The Nurses' Books.

Every district nurse should be provided with well arranged books so that she can keep a record of her work in a clear and methodical manner. If the association is affiliated with the Queen's Jubilee Institute or with a county association excellent books will be provided; if not, the association can get its own books printed on much the same plan, or if that expense must be avoided, ordinary exercise books do very well and the nurse can rule the lines herself. The books to be kept are: 1. Case book; 2. Time book; 3. Visit book. In the case book the nurse enters each case she attends, writing what is necessary under each heading, and it is a good plan to make a summary of the cases at the end of each month—Number of medical cases, number of surgical cases, number of maternity cases, number of chronic cases.

*Case Book.*

The nurses' case book should have 12 columns ruled, and extend across two pages. Particulars of several cases can be given in one opening of the book. The name and address, name of doctor, nature of disease, treatment, result, date of commencement and termination of the case, etc., should be recorded.

*Time Book.*

In the time book she keeps a record of the number of hours on duty and the number of visits paid each day. Some associations make the rule that "The nurse shall be on duty eight hours daily." It is better to say she "shall not be on duty longer than eight hours if possible," for at times the nurse is absolutely obliged to be on duty longer than eight hours, and at other times there may be so little work that she may only be on duty two or three hours. The nurse should as far as possible, however, be regular in her hours of going out and coming in, getting the bulk of her work done in the morning, resting in the afternoon, and seeing cases that need a second visit in the evening. In country districts in the winter it is often necessary to go out in the afternoon if there are long distances to go, so as to get finished before darkness comes on. In any case urgent calls should be attended to at once, no matter what the time of day. The number of visits are also entered in the time book each day, but it must not be thought that the number of visits paid is always a test of the amount of work done. The nurse may pay only six visits on one day, and be much busier than on another day when she has paid 12 or 13 visits; it all depends on the nature of the cases, how much she has to do for each patient, and the distances she has to go to their houses.

*Visit Book.*

In the visit book a daily record is kept of the number of visits paid to each patient, so that when a patient is taken off the books it is quite easy to reckon up the number of visits he or she has received. An exercise book with the lines ruled in squares is all that is required, each visit is marked with a stroke, or a C if it is only a casual visit, *i.e.* nothing to do for the patient, and a *X* may be put when the patient is taken off. Besides these three important books the nurse should keep account of any appliances, etc., lent out. These are generally lent free, but in some districts a nominal charge is made. The nurse is responsible for the safe return of any things thus lent, and should see that they are quite clean before putting them away. She may call this her "loan book" and an ordinary note-book will do.

(*To be concluded.*)

### Gynæcological Nursing.

The British Gynæcological Society held an examination for its certificate in gynæcological nursing on September 24th and October 1st, the written paper being taken on the former, and the *vivâ voce* examination on the latter date. Each candidate has a quarter of an hour's examination in practical nursing details by two matrons, and then a quarter of an hour's *vivâ voce* by the medical board of examiners. In the case of provincial candidates, and to save them the double journey to London, the written papers are answered under the supervision of hospital matrons—appointed by the Society for that purpose—in the various towns in which the candidates live. The successful candidates were:—Miss Margaret Anderson, Miss Helen M. Craig, Miss Martha Hood, Miss Pinkney, Miss Elizabeth Thompson, and Miss Bessie Wingfield. No candidate obtained the maternity nursing certificate on this occasion.

### The Written Questions.

The following were the written questions:—1. What is the cause of a ruptured perineum, and how would you prepare the patient for the necessary reparative operation, if this be performed six months or more after the accident? 2. What procedure would you adopt if directed to wash out the bladder? What solution is most often used for this purpose, and of what strength? 3. How would you prepare gauze-strips for packing the uterus, and how long are these usually left *in situ*. 4. What is a rigor, and what would you do for the patient who was suddenly seized with one? 5. How would you prepare the sponges or pledgets for an abdominal operation? 6. After an abdominal section, what variations in the temperature and pulse would you watch for, and what conditions would such variations severally denote?

### Nursing in the Netherlands.

### A VISIT TO THE GENERAL HOSPITAL, HAARLEM.

ALTHOUGH I had expected that a Dutch hospital would be in every way spick and span, I was not prepared for the very high standard of efficiency I found during a recent visit to the St. Elizabeths-Gasthuis, at Haarlem.

I called upon the matron, by appointment, and was courteously received by her. Miss Metelerkamp, the "Directrice," is a fully-trained and certificated nurse. Since May, 1902, she has been matron at Haarlem, but she has been nursing some twelve years. After her training at Amsterdam was completed, she studied at Heidelberg and Carlsruhe. Miss Metelerkamp went out to South Africa, and was in charge of a field hospital at Modderspruit during the Boer war. She also nursed at Ladysmith, Smaldeel, Brandford, Nooitgedacht, and other places.

"Yes," she said, "I nursed many of your soldiers—officers and men—who were prisoners. Most of the cases were typhoid. I was introduced to Lord Roberts, and saw several of your other great generals."

"I formed a very high opinion of your English hospital ships," she continued. "The one I went over at Durban gave me a good impression of the practical way your nursing was carried out in a hospital ship."

"May I ask if you have any medals or Orders?"

"I have a medal presented to me by our young Queen, in recognition of my services during the war in South Africa. I also hold a White Cross certificate for having passed a successful examination."

### The Nursing Staff.

Then, after a few exchanges of ideas upon the war itself, the field hospitals, and the "plague of women" out there who, though well meaning but amateur nurses often did more harm than good, we turned the subject of conversation.

"Will you please tell me about the nursing here?" I asked. "How many beds have you?"

"One hundred; and twenty-eight nurses. We have no ward 'Sisters' as you have in England—I have been over many of the London hospitals—but we have head nurses instead."

"And how long does the training take?"

"Three years. At the end of that time if the nurses have passed their examinations and been satisfactory in other ways they obtain their certificates for general nursing. They also qualify in monthly nursing during their three years, for we have a maternity department. The training here is really excellent. Some of the nurses go in for private nursing when they finish, others remain as head nurses in this hospital."

"Have the probationers salaries?"

"Yes, the first two years they receive 100 gulden (about £8 6s. 8d.) a year, and the third year 150 gulden. At the end of the third year those who remain on the staff and have passed their examinations get 300 gulden, with a yearly rise of 50 gulden till 500 are reached. Those who are not successful in their examinations can never be paid more than 200 gulden a year. I like the nurses to be about 25 years old at the time of training. They are all well educated, refined women, and are, of course, Protestants. We have two male nurses."

"What are the hours of duty?"

"For day nurses from 7 A.M. till 9 P.M., with one or two hours off duty daily, and ample time for meals. Night nurses are on duty from 9 P.M. till 9 A.M., and are never on night duty for more than a fortnight at a time."

"And holidays?"

"They all have a month a year, and every six weeks a whole 24 hours off duty."

"And with regard to your own duties?"

"Mine, of course, include the usual superintendence of nurses, the correspondence, and taking the head of the table at the nurses' meals. I always accompany the doctors on their morning rounds—we have two physicians and a house surgeon—and I am present at every operation, acting as anæsthetist. There is a housekeeper who entirely relieves me of the housekeeping department."

### The Wards and Patients.

Then Miss Metelerkamp took me over the hospital. The wards, not too large, are airy and extremely tidy and well kept. The box-like Dutch beds—resembling our children's cots—were covered with scarlet counterpanes, neatly folded. The ward tables, prettily arranged, the bright walls, the nurses themselves in their neat blue cotton uniforms—they wear aprons but no caps—must account a good deal for the happy expressions on most of the patients' faces. Everywhere relief, comfort, cleanliness, and method seemed to prevail. The children's ward looked particularly bright, and a large autographed picture of Queen Wilhelmina was in a prominent position. The maternity wards were conspicuous for their rows and rows of snowy swing-cots. The operating theatre looked well fitted and spotlessly clean.

The hospital is generally full, and every kind of disease gets treated there. There is no medical school attached so the nurses have all the dressings to themselves, under the superintendence of the house-surgeon.

The building itself is of great interest. It was once a monastery, and two of the rooms now used as board-rooms—the nurses have their examinations in one—are nothing less than museums. They are typical specimens of sixteenth century Netherland architecture and furniture; and the china, brass and pewter ornaments are of considerable value.

But, from an antiquarian point of view, the kitchen is perhaps the gem of the hospital. It is very large, and the walls are completely tiled with old Delft china; while racks and racks of most beautiful—all sixteenth century—Delft plates form a kind of frieze that seems wanton waste in a kitchen; and handsome antique "brasses" shine everywhere. The curios remain intact and where the monks left them.

If I were a nurse at the Haarlem Hospital I should pray that I might often be sent on errands to the kitchen; even to fail in an examination would be a good excuse for second visit to one of those treasure rooms.

### "The Hospital" Convalescent Fund.

THE Hon. Secretary begs to acknowledge with thanks the receipt of 2s. from Miss Walker.

### Everybody's Opinion.

[Correspondence on all subjects is invited, but we cannot in any way be responsible for the opinions expressed by our correspondents. No communication can be entertained if the name and address of the correspondent are not given as a guarantee of good faith, but not necessarily for publication. All correspondents should write on one side of the paper only.]

### A CAUTION.

"J. A. G." writes from Sydenham: I should like to warn superintendents of nursing homes to beware of a tall, thin lady dressed in black, coming (as she says) seeking a home for a sister who is expecting her accouchement in a few weeks' time. She came here, arranged to bring her sister that day or the next, accepted hospitality, and on leaving asked me to lend her money, which I very foolishly did. She said she had set off out with a friend and left her purse in her friend's bag as she had no pocket. She told me she was a Miss Norman, and was Miss Thorold's assistant (Middlesex Hospital), and as the latter had resigned the appointment had been given to her; that her father was a Chaplain to the Forces and a Canon of Windsor; and that another sister was matron at Netley. I have never heard another word from her, and I am afraid it is goodbye to the 7s. I lent her. I should know her anywhere if ever I met her. She talked of people I knew, and of doctors and nurses I have worked for and with. I certainly thought her genuine.

[It is a prudent rule to refuse to lend money to strangers, however plausible.—Editor, The Hospital.]

### THE ANGLO-AMERICAN NURSING HOME IN ROME.

"A Nurse," who states that she represented the nursing staff, writes: In connection with the various paragraphs which have appeared in The Hospital concerning "the unsatisfactory state of affairs" at the Anglo-American Nursing Home in Rome, I, together with five nurses who have returned and two others whom illness has prevented from returning, beg to state that during the whole season from October 1902 to July 1903 we had no occasion to appeal to the Committee on any grievance whatever in connection with the home. Surely this fact alone, of seven nurses out of eleven wishing to return, proves that the home must be on a more satisfactory footing than the general public are led to believe. The home is most comfortable and we are very happy, and no one is more anxious for the personal comfort and welfare of the nurses than the matron herself. There is no fear that the nursing home will fail for lack of nurses, considering that over 100 applications were received by the matron from nurses in England and America for this season. In every sphere of life there are always some who are inclined to make mountains out of molehills, and the exaggerated letter of the "German" nurse in your edition of September 5th is about as true as her statement at the beginning in calling herself "An English Nurse." I trust that you will kindly insert this in justice to the home. I beg to enclose my card on behalf of "The Nursing Staff."

### MIDDLE-AGED NURSES.

"One of the Unfortunate (40)" writes: In the current number of The Hospital there is an article on the 40-year-old nurse and what to do with her. I could not help thinking when reading it how very hard it must appear to many of your readers. According to the present rules a woman cannot enter a training school till she is 23 at the earliest; allowing three to four years in which to train would make her 27 when she starts life as a "trained nurse." All cannot be matrons, however excellent their attainments may be, yet after all the toil, expense, and time given, a nurse is to be pushed aside as useless at 40, worn out from start to finish in 17 years! Allowing that some wear out quicker than others, is it an inducement to enter the nursing world to know that you may be able to get an appointment at 40 as a "cottage mother," if all things are equal? I think the nursing world is rather crowded at present; what is to become of them all at 40? It is a well-established fact that very many nurses work to help some member or members of their family. How is it possible for them to save and make a provision for themselves under such circumstances? Some have no ties, and work for themselves, and they, by belonging to a nursing home, may receive from £25 to £35 a year. Do you think the public pay so liberally that nurses can save in 13 years (the very longest time at their disposal) enough to keep them, say 20 years? The Royal National Pension Fund is a very excellent thing but you must pay in before you can draw out, and unless I have read incorrectly, you cannot have a pension till 45, and even then your payments must be large or you can only draw, say, £20 a year; will that amount keep and clothe as well as provide a home for a woman? We are at the other end of the pendulum. A few years ago anyone under 40 was reckoned too young for a responsible position. It may be, however, that nursing the sick and suffering is not considered a responsible work and experience not required? I should be glad to know what others think on the subject, for our daughters ought to be warned not to enter the nursing profession. Forty years of age is late for a woman to begin to turn her attention to a new occupation after having given the best of her life to acquire the efficiency so absolutely necessary for a successful trained nurse.

### THE CLOTHES OF MEDICAL MEN.

"Sister" writes: It seems to me that it is rather a mean thing to criticise medical men in any way, as I have the greatest admiration for them; but there is just one inconsistency of theirs I would like to bring to their notice. Nowadays who has not heard of the haunting and irrepressible microbe? Some of us who are of a nervous disposition dread him more than enough. We take the utmost precautions for his destruction. We drink no unboiled water, our milk is sterilized, we use disinfectants innumerable, but still the insidious foe creeps in and attacks us unawares, now and then, in spite of all our efforts. If any of our family are attacked by a dread "microby" disease, what infinite care our good "medico" takes that it shall not spread to anyone else, and, if we are wise, we follow out all his directions with the greatest zeal. But as for thinking one moment of himself, no doctor, worthy of the name, would dream of doing so. A good many doctors fall victims to the diseases they catch from their patients, but do they get any special thanks for it? Oh, no! They have only done their duty; they are perhaps invalided for life, or die outright, and their place knows them no more, and that is all. The flourishing and busy physician sallies forth for the day's work in his well-appointed brougham, attired in an immaculate suit of broadcloth, looking prosperity itself, from the glossy crown of his silk hat to his beautifully polished boots. But then such nice, well-fitting clothes cost money, they are not to be lightly thrown on one side and discarded. Now our physician has to enter all sorts and descriptions of sick rooms, swarming with many and various bacteria, microbes, germs, etc. He has to rub up against bedclothes, on which, if they are as numerous as scientific people tell us, bacilli must be settling in hundreds. The consequence is that a few must, willy nilly, cling to the handsome coat and irreproachable trousers, the doctor conveys them home with him unconsciously, and that nice, respectable-looking suit of clothes may become shortly a very field of action for fighting and struggling microbes. If the medical man were attired in a clean white washing suit when he went on his rounds, he could consign it to the wash tub on his return, where the microbes would be boiled out of existence, and every day he could put on a fresh clean suit. Think of the economy, too. On cold days he could wear out his oldest things safely hidden under his white washing attire. Also, how becoming white is to the majority of Britishers, and how smart our medical men would look attired in white (with perhaps a white yachting cap) driving their spick and span dog-carts. I hope the medical men will not be offended at this little suggestion, but will take it in friendly and good part as it is meant.

### Appointments.

[No charge is made for announcements under this head, and we are always glad to receive, and publish, appointments. The information to insure accuracy should be sent from the nurses themselves, and we cannot undertake to correct official announcements which may happen to be inaccurate. It is essential that in all cases the school of training should be given.]

Cumberland Infirmary, Carlisle.—Miss F. M. Hodges has been appointed night sister. She was trained at the Bristol Royal Infirmary, and has since been sister for two-and-a-half years.

Derby Royal Infirmary.—Miss E. Bosher has been appointed sister. She was trained at Monsall Fever Hospital, Manchester, and at Leeds General Infirmary.

Incorporation Infirmary, Shirley Warren.—Miss Mary C. Byrne has been appointed assistant matron and superintendent of the nursing home, and Miss M. Cicely Elmhirst has been appointed night superintendent. Miss Byrne was trained at St. George's Infirmary, Fulham Road, London, subsequently holding the post of sister. She has since been night superintendent at the Woolwich Infirmary, Plumstead. She holds the L.O.S. certificate. Miss Elmhirst was trained at the Royal Alexandra Hospital, Rhyl, and was attached to the private staff of that institution until 1898. She afterwards joined the private staff of Princess Christian's Nurses' Home at Windsor, and later received training at Chelsea Infirmary. She has since held the post of ward sister at Shirley Warren Infirmary.

Passmore Edwards Hospital, East Ham.—Miss Dorothy Bracewell has been appointed sister. She was trained at St. Bartholomew's Hospital, Rochester, and has since been sister at Friedenheim, Swiss Cottage, London.

Rhymney Workmen's Cottage Hospital.—Miss Mary J. Matthews has been appointed nurse-matron. She was trained at Newport (Monmouthshire) Infirmary and St. Marylebone Infirmary, London. She was subsequently nurse at Llangollen Cottage Hospital, head nurse at Llanelly Hospital, South Wales, and staff nurse and sister-in-charge at the Throat Hospital, Golden Square, London.

Royal Naval Hospital, Plymouth.—Miss Kathleen E. Key Swindale has been appointed nursing sister. She was trained at St. Bartholomew's Hospital, London.

South Wimbledon Cottage Hospital.—Miss A. Agatha Hilliary has been appointed staff nurse. She was trained at Gravesend Hospital, and has taken temporary matron's duty at Wimborne Cottage Hospital.

Swansea Hospital Convalescent Home.—Mrs. S. A. Gamwell has been appointed matron. She was trained at Swansea Hospital and the London Hospital. She has since been matron of Swansea Borough Hospital, and has also done private nursing.

### Presentations.

St. Mary (Islington) Infirmary.—On resigning her post as assistant matron at St. Mary (Islington) Infirmary, Highgate, Miss Eyles was presented with a very handsome cherub choir silver hand-glass from the nurses as a token of love and sincere regard, and a black morocco dressing case with celluloid fittings from the laundry hands as a token of sincere regard and respect, with two very pathetic verses composed by one of the women. Miss Eyles takes with her the goodwill of all.

Dundee Royal Lunatic Asylum.—Miss Annabel Irvine, on relinquishing her appointment as matron of Dundee Royal Lunatic Asylum, has been presented by the nursing staff with a handsome silver tea service. Miss Irvine afterwards entertained the staff at supper.

### The Nurses' Bookshelf.

The Private Nurse's Own Notebook. By Sister Eva. (Published by Bemrose and Sons, London. Price 6d.)

This is a simple little book in paper cover designed for the nurse's own use, to record a brief outline of each case she attends, and the expenses incurred and money received in connection with it. As the book is to be retained it appears to us that a more durable binding would have been better.

Nursing Notes on Midwifery and Gynæcology. By Sister Ross. Pp. 134. 12mo. Price 2s. (London: The Scientific Press, Limited. 1903.)

The notes of which this useful little book is composed were written at first for the use of the nurses connected with the Rotunda Hospital, Dublin. They make no pretension to anything new or elaborate, but undoubtedly will be helpful to nurses during their early days of training, and to nurses engaged in private work, if used as stepping-stones to larger and more ambitious books. The statements are dogmatic, of course, but they are accurate. The language is clear and simple, and little points are insisted on that would perhaps escape a man's observation, but yet would strike a nurse as important, and very justly so. Sister Ross has accomplished her task admirably, and has shown the faculty of imparting to others the knowledge of essentials.

Motherhood: The Wife and Mother: A Book of First Principles for the Guidance of Young Married Women. By Ralph Vincent, M.D., M.R.C.P. (London: The Walter Scott Publishing Company, Limited. 1902. Price 5s.)

Since Eve played with her grandchildren women have not lacked "advisers" regarding the management of motherhood. The volumes claiming to be safe guides through the perils and perplexities of childbearing are simply legion. Women have a wonderful gift of doing what they should not do and leaving undone what they should do when overtaken by pregnancy; and at such a time they are only too apt to fall victims to superstition and ignorance. The present work, in spite of its unsatisfactory title, aims at providing reliable guidance and safe direction to the recently married woman. But Dr. Vincent's reader should belong to the well-to-do class if she is to avail herself of his suggested "pregnancy corset-belt" and enjoy the benefits of massage during the lying-in. We fear practitioners labouring in the East End or toiling amongst the poor in manufacturing and agricultural districts will see a humorous side to many of the author's directions. There is much in this little work that is excellent. An endeavour has been made to prevent readers from relying on book reading instead of medical advice; and drugs are with but few exceptions not mentioned. Dr. Vincent is not judicious when he says "good brandy is the best stimulant, when immediate stimulation is required, but it should be reserved for the necessary occasion," for it is generally recognised now that a healthy pregnant woman is best without alcohol in any form. Many will take exception to the author's contention that "the treatment of a case of labour without chloroform is, in my opinion, an inhumane and unscientific proceeding," and we really think Dr. Vincent cannot be congratulated on his anticipation of "the time when anæsthesia will be regarded as essential in these cases as it is in surgical operations." We also take exception to certain statements concerning the "disadvantages of breast-feeding." The upper-class woman of to-day needs rather to be told something of the advantage and duty of nursing. But in spite of not a few sins of omission as well as commission, Dr. Vincent's little work is likely to serve a useful purpose, but we should much like to be able to see the author's revised version of it 50 years hence.

### Echoes from the Outside World.

### Movements of Royalty.

The King returned to London on Thursday last week in excellent health, and was loudly cheered by the people along the route from Euston Station to Buckingham Palace. On Friday morning his Majesty held a Council in connection with the reconstruction of the Cabinet, and subsequently a Royal Proclamation was issued announcing the further prorogation of Parliament from November 2nd, the original date, to Friday, December 11th. On Saturday evening the King and the Prince and Princess of Wales—who also returned to London last week—visited His Majesty's Theatre to see the performance of "Richard II.," occupying a private box abutting on the back of the stalls which had been specially fitted up by Mr. Tree so that a direct and uninterrupted view of the stage might be commanded. On Tuesday the Prince and Princess of Wales visited the Whitechapel Art Gallery, and spent more than an hour in examining the collection of models and pictures in the Shipping Exhibition.

### The Reconstructed Government.

Several further changes have been made in the Government. Lord Londonderry becomes Lord President of the Council. Lord Salisbury, better known as Lord Cranborne, has relinquished the office of Under-Secretary for Foreign Affairs, only to enter the Cabinet as Lord Privy Seal, a position which his father held before him. His place at the Foreign Office is taken by Earl Percy, heir to the dukedom of Northumberland, and the important office of Secretary to the Treasury has been accepted by Mr. Victor Cavendish, heir to the dukedom of Devonshire. Mr. E. G. Pretyman is promoted to be Secretary to the Admiralty, and Mr. A. H. Lee, member for the Fareham Division of Hampshire, becomes Civil Lord of the Admiralty. Mr. Bromley Davenport, M.P. for the Macclesfield Division of Cheshire, joins the Government as Financial Secretary to the War Office; Lord Balcarres, M.P. for the Chorley Division of Lancashire, as Junior Lord of the Treasury; and the Marquis of Hamilton as Treasurer of the Household. Lord Hamilton is heir to the dukedom of Abercorn, and nephew of Lord George Hamilton, who recently left the Cabinet.

### Russia and Japan.

On Tuesday alarming reports were current respecting the relations between Russia and Japan, but it has since been stated by the Japanese Minister in London that he has received reassuring news from Japan in reference to the situation in the Far East. The Japanese Minister in Paris affirms that Japan entertains the most cordial diplomatic relations with Russia and that no tension exists between the two countries, while the Japanese Minister in Berlin declares that there is no cause whatever for apprehensions of an outbreak of war.

### The Church Congress.

On Tuesday the second Church Congress held in Bristol was opened. The first took place in 1864, and a feature of the present meeting was that Bishop Ellicott, who, as Bishop of the undivided See of Gloucester and Bristol, presided on that occasion, was on the platform. The President of the Congress this week is, of course, the Bishop of Bristol, who before his appointment to the see, was well known as Canon G. F. Browne, of St. Paul's Cathedral. Congress sermons were preached at the Cathedral by the Primate, at St. Mary Redcliffe by the Bishop of Worcester, at Clifton Parish Church by the Bishop of Winchester, and at All Saints', Clifton, by the Bishop of Truro. The President's address was delivered in the Colston Hall on Tuesday afternoon. He referred to the historical fact that the first Church Congress was held 1,300 years ago within the border of the diocese of Bristol, as an illustration of the wonderfully interesting history of the National Church of England. He maintained that no national Church had so few episodes in its history for which men of the present day had to blush. Having urged that the Church must always manage its affairs in a broad English way, and never again admit the management of a foreign Church, Dr. Browne contended that Churchmen must be free, within the limits of certain ruling principles, to expand and to adapt their work to its changing conditions and surroundings. The Congress then appropriately proceeded to consider the subject of "Variations in a National Church."

### The New Bishop of Manchester.

The King has approved of the appointment of the Right Rev. Edmund Arbuthnot Knox, Bishop Suffragan of Coventry, as Bishop of Manchester, in succession to Bishop Moorhouse. Dr. Knox, who is the son of a clergyman, was born at Bangalore, India, where his father was a chaplain to the East India Company in 1847. In 1868 he was elected Fellow of Merton College, Oxford, and in 1870 he entered holy orders and for four years was voluntary curate of Holy Trinity, Oxford, after which he was Vicar of St. John the Baptist in that city. In 1884 he accepted the college living of Kibworth, Leicestershire, and in 1891 he became Vicar of Aston, Birmingham. When he went to Aston there were four Sunday schools in connection with the church, and when he left, at the end of three years, there were fourteen, with an average attendance of upwards of 2,000 children and 200 teachers. In 1894 he became Bishop Suffragan of Coventry under Dr. Perowne, then Bishop of Worcester, and a year later rector of St. Philip's, Birmingham. For some years Dr. Knox has greatly interested himself in the question of education, and the promotion of the Birmingham bishopric. His unselfish devotion to his labours, his kindly disposition, and his scrupulous fairness to all parties, have rendered him extremely popular.

### Regimental Cottage Homes.

The Royal Berkshire Regiment has received from Mrs. Lewis, in memory of her late husband, Mr. S. Lewis, a gift of £3,000 for the purchase of a site and for building and endowing in perpetuity three cottage homes for disabled men of the regiment with their families. The homes, situated near the barracks at Reading, are now nearly completed. They are well planned and picturesque, with high-pitched gabled roofs, and there is a good-sized garden attached to each of them. The timely gift of Mrs. Lewis will doubtless act as a stimulus to others to help in the good work of the Regimental Cottage Homes Fund.

### Mr. Pinero's New Play.

A new play called "Letty," by Mr. A. W. Pinero, was produced at the Duke of York's Theatre last week, with every manifestation of success. Mr. Pinero has almost exclusively portrayed in this clever drama men and women belonging to the shady side of life, with an abundance of faults and few virtues. There is nothing original nor remarkable in the story, which has been told over and over again, but the dialogue is very bright and amusing, and the details of the scenes, like the sentiments of the persons in the play, are, no doubt, faithful representations of facts and individuals as the author has seen them. The picture of a low-class City man, who calls himself an outside broker, is by no means overdrawn. The fate of the heroine, if she merits that title, has the advantage of being more in accordance with what happens in real life than in trashy novels. The acting all round is good. Miss Irene Vanbrugh in the character of Letty, Mr. H. B. Irving as the scoundrel who is supposed to be a gentleman, and others do full justice to the parts allotted to them.

### A Book and its Story.

NEW NOVEL BY MRS. CROKER.*

Mrs. Croker's new story of Irish peasants, varied by some experiences taken from life, of the hero, Shamus MacCarthy, is bright and interesting, as her stories invariably are. Tears and laughter, comedy and tragedy, are mingled in a delightfully Irish manner. Johanna is a typical Kerry girl, growing to womanhood in the isolated valley where the farm, which, with some adjoining acres, formed a little property belonging to her father, John Daly, was situated. His description is the reverse of attractive. "He was a sour-faced, elderly widower, an active, hard-working man, terribly fond of money, who would beat the devil himself at a bargain. John's cabin was situated in a lovely but lonely spot, so isolated as to be extremely inconvenient for his trade; but as it had been in his family for several generations, John Daly would as soon have contemplated changing his religion as his residence. He owned a banking book, for he was no believer in teapots and stockings, which would have opened the eyes and mouths of his acquaintances had they been privileged to glance at its contents." He was possessed also of some household treasures, a fine dresser of crockery and an eight-day clock. He had also a "companion and confederate" in a long black dog which answered to the name of "Pup," and who accompanied him on all expeditions, whether to collect eggs, or to snare, or shoot a hare or rabbit—for John Daly was an inveterate poacher. "The 'Pup' had yellowish eyes, and if you looked into them steadily, you encountered a gaze of bland benevolence, tempered with extraordinary cunning and self-esteem." As a young man Daly had married, and brought home, a beautiful Irish girl from beyond the Shannon. "She was terribly stand-off in herself, and made no freedom with her neighbours, and perhaps, in consequence, died young." She left, as a legacy to the dour-faced husband, her child Johanna, and she grew up, serving her father with patience and industry. The cottage home, "whitewashed by Johanna's capable hands," stood on rising ground, surrounded by a little garden full of monthly roses, fuschias, and marigolds. Johanna's kitchen reflected her own love for the beautiful in its arrangements. There were flowers—"exquisite pink-tinted water lilies stolen from the bosom of a tiny loch that lay in the purple lap of a hill a mile from the cabin"—standing in a tin porringer that shone like silver; they gave a touch of beauty and refinement to the homely interior. Without, the eyes of Johanna met this lovely scene. "Underfoot is deep, soft moss on one side lies the short-cropped turf and vivid stretches of furze; on the other, the anxious river gleams and foams over the grey pebbles. Above, hang clumps of wild fuschia; while overhead are dark mountains and the soft blue sky."

And Johanna herself—here she stands for our inspection. "Goddess of the loch, glen and mountain. Twenty years of age, five feet ten inches in height and splendidly handsome, wearing a blue cloth cape, thrown over her shoulders as if it were a royal mantle. . . . Her eyes are a deep grey, her hair, which is wound round her head in massive coils, is as black as night. Her features are not small, but they are perfectly modelled, her brow is broad and low, the chin rounded, her upper lip short, the skin, where not sunburnt, is dazzling fair. Her throat and arms are white as milk." Johanna had no neighbours. She grew up in ignorance of many things, the magnetic influence of beauty among others. Her only companions and friends were the creatures on the farm that were attached to her with the affection of dumb animals. "A couple of little Kerry cows and a brace of boneens, who followed their mistress, partly from pure affection and partly from the passionate craving to see and be seen which is so deeply implanted in the heart of every Kerry pig." Johanna was just an honest, self-contained, pious girl. She believed in her parish priest and St. Joseph, and adored the Blessed Virgin, and Shamus MacCarthy. But thanks to her industry and ignorance she had little time or inclination to go abroad, and it was only in the little white-washed chapel on Sunday that the outside world beheld the Beauty of the Valley. Then, "little did she guess, as she knelt in prayer, with her hood over her head and her beads in her hand, that many and many a boy had walked miles over the hills or up from the shore just as much to get a look at Daly's daughter as to hear the mass."

Marriage among the peasantry in Kerry is more a matter of business than outsiders are aware. "They are invariably arranged between the parents of the boy and girl with the aid of a professional 'go between,' or 'Fostooke.' In weighing all the pros and cons, with the accompaniment of shouts, protestations, invocations, and a liberal supply of porter—the claims of a stout jennet—or even a flock of geese—have a better chance of consideration than such ridiculous balderdash as love at first sight; or, indeed, any love at all!" Johanna, so far, had had but one suitor, and he not at all to her mind. John Daly knew the value of his daughter, and what her marriage would mean to him. "Who would mind the house, and dig, and milk, and work as she did? Moreover, her earnings came to a considerable amount. He pocketed all the fowl and butter money, and half her profits on every piece of tweed." The village matchmaker was in despair, for the fixing up of a match meant a substantial recognition of her services. One afternoon she strayed casually in the direction of the Daly's cabin, and encountered Shamus MacCarthy. "Shamus McCarthy with his curly head and impudent blue eyes, swinging his stick and whistling like a blackbird." He was the last person that she wished to encounter at this particular moment, as her design was to waylay Johanna's father, with a second offer for Johanna from Timothy Laffan, the contractor. He was also owner of a public house in the neighbourhood. Timothy was the suitor recently rejected with scorn by Johanna.

"Now it had been whispered that Shamus was the only boy who had ever received the smallest encouragement from Johanna. He was a handsome fellow, well made, and graceful and lithe as a greyhound, active as a cat. He had a pleasant word and a joke for man, woman or child, and he could dance down any boy in the barony. Besides all these attractions . . . he had a smile that would coax the birds out of the bushes." There was another side to this fascinating description. "It must be confessed that Shamus had the reputation of being a 'bit wild,' and fond of fighting—as well as a sup of porter, and the girls—and many a girl was fond of Shamus. Some he courted in public and some on the sly—and, alas, among these latter was Johanna Daly." Shamus gets mixed up in a fight one night, and although he was more sinned against than sinning, he bolts and enlists. Previous to this he had secreted himself from his enemies. This is the parting scene between the lovers. "Shamus' handsome young face was gaunt with privation and anxiety. He took the beads which hung loosely from Johanna's hands, and kissed the little cross with impassioned devotion. Then he held Johanna for one moment in his arms, sprang over a low stone wall, and was gone like a dream." After much suffering on the part of Johanna, and much active service on his own, Shamus returns one day, and all ends happily.

* "Johanna." By Mrs. Croker. (Methuen. 6s.)

### For Reading to the Sick.

AUTUMN.

With what a glory comes and goes the year?
The buds of spring, those beautiful harbingers
Of sunny skies and cloudless times, enjoy
Life's newness, and earth's garniture spread out.
And when the silvery habit of the clouds
Comes down upon the autumn sun, and with
A sober gladness the old year takes up
His bright inheritance of golden fruits,
A pomp and pageant fill the splendid scene,
Pouring new glory on the autumn woods,
And dipping in warm light the pillared clouds.

O what a glory doth this world put on,
For him who with a fervent heart goes forth
Under the bright and glorious sky, and looks
On duties well performed, and days well spent!
For him the wind, ay, and the yellow leaves,
Shall have a voice, and give him eloquent teachings.
He shall so hear the solemn hymn, that Death
Has lifted up for all, that he shall go
To his long resting-place without a tear.

*Longfellow.*

Autumn is here, and it is already late. He has painted the hedges russet and gold, scarlet and black, and a tangle of grey; and he has damp brown leaves in his hair, and frost in his finger-tips.

It is a season of contrasts; at first all is stir and bustle, the ingathering of man and beast; barn and rickyard stand filled with golden treasure; at the farm the sound of threshing; in wood and copse the squirrels busied 'twixt tree and storehouse, while the ripe nuts fall with thud of thunder rain. When the harvesting is over, the fruit gathered, the last rick thatched, there comes a pause. Earth strips off her bright colours and shows a bare and furrowed face; the dead leaves fall gently and sadly through the calm, sweet air; grey mists drape the fields and hedges. The migratory birds have left, save a few late swallows; and as I sit at work in the soft, still rain, I can hear the blackbird's melancholy trill and the thin pipe of the redbreast's winter song—the air is full of the sound of farewell.

Forethought and preparation for the Future which shall be; farewell, because of the Future which may never be—for us; "Man, thou hast goods laid up for many years, and it is well; but, remember, this night thy soul may be required;" is the unvoiced lesson of autumn.

It is manly, not morbid, to dare to taste one pungent savour of pain, the lingering sadness of farewell which emphasises the aftermath of life; it should have its place in all our preparation as a part of our inheritance we dare not be without.—*M. Fairless.*

Come then with all thy grave beatitudes
Thou soother of the heart and of the brain
Autumn! whose ample loveliness includes
The pleasure and the pain

Of all that is majestic in despair
Or beautiful in failure. Hast thou failed?
The winds of heaven among thy branches bare
Have wrestled and prevailed.

*A. Munby.*

### Notes and Queries.

FOR REGULATIONS SEE PAGE 21.

*Hospital Training.*

(25) Would my height, five feet, prevent my being a nurse? I am 24, strong, and well educated. Kindly give me the names of any English or Irish training schools.—*E. J. T.*

Your height is certainly against you; but consult "The Nursing Profession: How and Where to Train," and if you find a difficulty try some of the training schools under the Local Government Board; apply, stating full particulars.

*Compensation.*

(26) Will you kindly say if a patient can dismiss his nurse after having several times asked her to stay, and having put her to the expense of furnishing room? There is no written agreement.—*Nurse T.*

The nurse ought to have had a definite agreement in writing before going to the expense of furnishing a room. There is not sufficient cause for making a legal claim for compensation.

*Epileptic.*

(27) I am a poor person, and I have a daughter eighteen years of age who is subject to epileptic fits. Is there a place where she could be placed under the care of a doctor who understands this disorder, free, or on the payment of a small sum?—*Her Mother.*

Apply to the Secretary, the National Hospital for the Paralysed and Epileptic, Queen's Square, Bloomsbury, W.C., and to the Meath Home of Comfort for Epileptics, Godalming.

*Rheumatoid Arthritis.*

(28) Could you tell me the name of a hospital in London for a patient suffering from rheumatoid arthritis?—*A. B.*

This is a point on which you should consult your doctor.

*Fever Training.*

(29) How can I obtain one year's special work in fever?—*Nurse.*

Apply the Secretary of the Metropolitan Asylum Board, Victoria Embankment, E.C.

*A Trade.*

(30) Can you tell me of any institution where a man partially paralysed could be taught a trade, such as brush or basket making. In or near Warwick preferred?—*M. C.*

Is there no local firm to which he could be apprenticed? Perhaps the Secretary of the National Industrial Home for Crippled Boys, Wright's Lane, Kensington, W., might be able to advise you.

*Hospital Training.*

(31) Will you tell me if there is any hospital at Brighton or at any seaside place, large enough to satisfy the requirements of the Local Government Board, where I could be trained?—*Sagittarius.*

The Sussex County Hospital, Brighton, and other seaside places.

*Home.*

(32) Can you kindly tell me of a home, or institution, which would be likely to receive my father? He has been paralysed for 12 years, and has been nursed at home, but my mother's health has failed, and she is now too ill to wait on him herself.—*A. M. K.*

A home for incurables would be suitable. Consult a list such as you will find in "Burdett's Hospitals and Charities."

Will you kindly tell me of a good home where a young woman suffering from epilepsy could be received? She is very fond of children and of needlework. Her friends could afford to pay a small sum for her weekly.—*Nurse M.*

The Meath Home of Comfort for Epileptics, Godalming.

**Standard Nursing Manuals.**

"The Nursing Profession: How and Where to Train." 2s. net; 2s. 4d. post free.

"Nursing: Its Theory and Practice." (Revised Edition). 3s. 6d. post free.

"Elementary Anatomy and Surgery for Nurses." By William McAdam Eccles, M.D.Lond., M.B. 2s. 6d. post free.

"Elementary Physiology for Nurses." By C. F. Marshall, M.D., B.Sc., F.R.C.S. 2s. post free.

"Ophthalmic Nursing." By Sydney Stephenson, M.B., F.R.C.S., 3s. 6d. net; 3s. 10d. post free.

"Nursing in Diseases of the Throat, Nose, and Ear." By P. Macleod Yearsley, F.R.C.S.Eng., M.R.C.S. 2s. 6d. post free.

"Fevers and Infectious Diseases." 1s. post free.

